# Pencil and Gold Electrode Materials for the Electrochemical Study and Analysis of Dinitrotoluene

**DOI:** 10.1021/acsomega.3c08741

**Published:** 2024-02-12

**Authors:** Kevin J. Kurian, Julie De Maere, Benjamin Schazmann

**Affiliations:** † Applied Electrochemistry Group (AEG), FOCAS Research Institute − Technological University Dublin, Aungier Street, Dublin 8, Ireland; ‡ Odisee University of Applied Sciences, Technology Campus Ghent, Gebroeders de Smetstraat 1, 9000 Gent, Belgium; § School of Chemical and BioPharmaceutical Sciences, CQ 441, Technological University Dublin, Grangegorman Lower, Dublin 7, Ireland

## Abstract

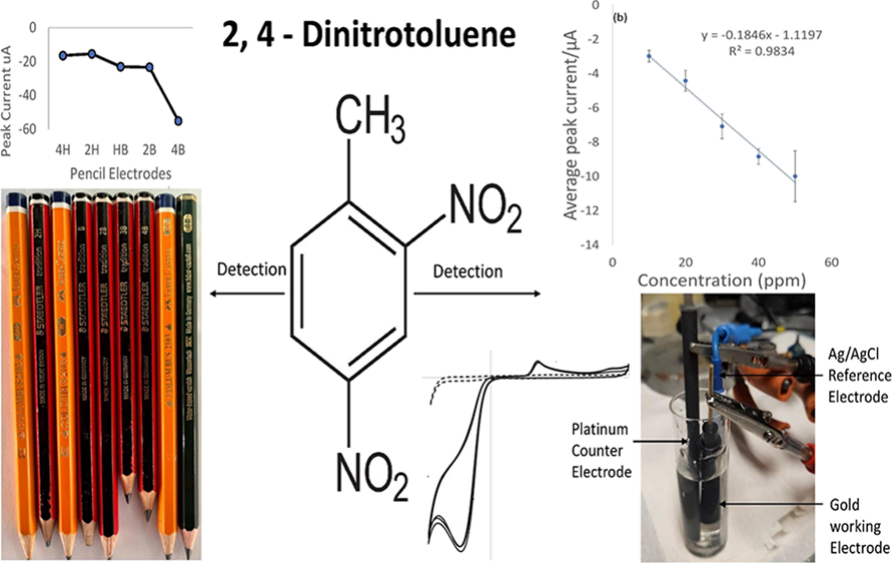

The aim of our work was to investigate practical and robust methods for the electrochemical analysis of DNT. Using gold WEs, we differentiated between the nitro substituents in 2,4- and 2,6-DNT in organic electrolyte systems. Switching to an aqueous electrolyte (2 M H_2_SO_4_), a limit of detection (LOD) of 0.158 ppm (0.87 μM) and a limit of quantitation (LOQ) of 0.48 ppm (2.64 μM) were observed for 2,4-DNT. Subsequent simplification to wooden craft pencils as WEs in aqueous 2 M H_2_SO_4_ electrolyte achieved a LOD of 4.8 ppm (26.48 μM) and a LOQ of 14.6 ppm (80.54 μM) for 2,4-DNT. Alongside this easily renewable WE choice, 2 M H_2_SO_4_ was found to improve the solubility of DNT in aqueous media and has not been previously reported as an electrolyte in DNT electroanalysis. On testing a range of pencil grades from 4H to 8B, it was found that 4B gave the best sensitivity. The work serves as a preliminary study into materials that, through their simplicity and availability, may be suitable for the development of a robust and portable instrumental method through the electrochemical work presented here.

## Introduction

Dinitrotoluenes (DNT) are precursors employed in the manufacture of many products such as ammunition, explosives, dyes, herbicides, plastics, elastomers, and coatings.^1,2^ They are very toxic to animals and humans where prolonged exposure has been reported to form tumors in the former and methemoglobinemia in the latter.^3^ The International Agency for Research on Cancer (IARC) classifies 2,4-and 2,6-DNT as Group 2B carcinogens (possibly carcinogenic to humans) and The American Conference of Governmental Industrial Hygienists (ACGIH) has classified the 2,4-/2,6-DNT mixture as an A3 carcinogen (confirmed animal carcinogen with unknown relevance to humans). Any level of exposure to carcinogens is considered unsafe.^4^

The presence of DNT in ammunitions and explosives makes it readily found in war zones such as in the Middle East, Africa, and increasingly in Europe. Difficult access and the frequent absence of a conventional laboratory infrastructure mean that simple and portable analytical methods are needed. Field-deployable testing would be quite advantageous and essential for these areas.^5^ The presence of these compounds is widespread in soil and water due to military activity and factory waste from other DNT applications, where it remains due to its very slow biodegradation. This over time leads to bioaccumulation and seepage into water streams, affecting other animals and plants, further harming the natural ecosystem.^6^ Thus, efficient and rapid methods of analysis, including field-deployable systems, are essential.

Current state-of-the-art DNT analysis typically uses various forms of mass spectrometry or optical spectroscopy, which require bulky, expensive instrumentation, large sample quantities, advanced methodologies, and modern laboratory infrastructure.^7^ Analysis times are relatively long, and well-trained personnel are required. These attributes may not apply where the need for DNT analysis is the most pressing.

The electroactive redox activity of the nitro groups in DNT provides the basis for the development of electrochemical sensors, which when compared with other analytical techniques provide simple, rapid, selective detection using relatively low-cost instrumentation.^7^ They also have many advantages such as broad linear dynamic range, cost-effectiveness, high accuracy, low limit of detection, direct determination of analyte, rapid response, and miniaturization.^8^ Current electrochemical sensors for 2,4-DNT reported in the literature often utilize complex modifications to the electrode surface and electrode (surface) renewal, alongside electrolyte systems that differ substantially from the sample matrix.^9−13^ Electrode replacement, reuse, and reduced sample processing for direct analysis in the field remain challenging. The use of complex nanocatalysts typically improves the detection of the analyte by increasing the dissociation and transport of charged particles, while also increasing the electrocatalytic activity. Further information on nanocatalysts is available.^14−18^ These modifications are expensive and laborious, creating hurdles for further field deployment. In answer, this paper suggests the use of simple bare electrodes in an uncomplicated close-to-sample-matrix aqueous electrolyte, with the analysis carried out readily by means of the ubiquitous cyclic voltammetry (CV) mode which is compatible with most portable potentiostats. This technique is very popular for studying the initial electrochemistry of new systems and provides substantial information for electrode reactions. It mainly operates by stepping up the working potential in one direction linearly versus time, and after a set potential has reached, the potential is reversed to the initial potential, providing information on electrochemical oxidation and reduction.^19^ Further basics of cyclic voltammetry can be read at refs (20−23). The work seeks comparable analytical limits of detection (LODs) to the literature, while looking into the electrochemical reduction mechanism of DNT, all contributing to analytical method optimization.

Pencil graphite electrodes in the recent few decades have been seen to be quite advantageous in the electrochemical detection of various organic and inorganic compounds, owing mainly to its benefits in mechanical resistance, affordability, and ready availability.^24^ These pencils are based on nanocomposite graphite with the intercalation of clay particles. The electroactive properties of pencils are due to their main component being graphite, which is made up of layers of graphene having extensive electron delocalization.^25^ This makes it a conducting material and suitable for use in electrodes. Pencil graphite electrodes have been used for the analysis of various nitroaromatics such as nitrobenzene, nitrophenols, and TNT.^26−35^

Electrochemical sensors require the analyte to be in solution, which in practice is difficult due to the low solubility of DNT in aqueous solvents. This becomes critical, for example, when extracting DNT from soil samples, potentially alongside other analytes of interest. It has been reported by Kong et al., however, that acidic solutions improve the solubility of DNT, and sulfuric acid is reported to have a reasonable potential window.^36^ Thus, sulfuric acid was explored as a supporting acidic electrolyte in the current work. Further, sulfuric acid is used as a general solubilizing agent in soil extraction processes and can potentially reduce the sample pretreatment required for electrochemical analysis after extraction.^37^

The aim of this work is to explore, for the first time, the use of pencil graphite and gold electrode materials in conjunction with a sulfuric acid solvent and electrolyte for the study of DNT in aqueous samples.

## Experimental Section

### Chemicals

2,4-Dinitrotoluene 99.8%, 2,6-dinitrotoluene 99.8%, and sodium perchlorate (all of analytical grade) were purchased from Sigma-Aldrich and used as received. The other chemicals used were acetonitrile 99.8% HPLC grade and concentrated sulfuric acid obtained from Fisher Scientific. Nitrogen gas was obtained from Air Products Irl Ltd. Solutions were prepared by using ultrapure water (18.2 MΩ cm). Sulfuric acid was used as the electrolyte in aqueous media, while sodium perchlorate was used as the electrolyte in organic solvents.

### Instrumentation

Voltammetric measurements were performed on an Eco Chemie B.V. Electrochemical workstation, model Autolab PGSTAT 12 (The Netherlands) using GPES software (version 4.9). All measurements were conducted using a three-electrode one-compartment configuration, where the working electrodes were either gold with a geometric diameter of 2 mm or seven pencil electrodes 4H (least graphite) to 8B (most graphite) from the Faber Castell 9000 commercial stationary series. The counter electrode was a carbon rod, and the reference electrode was a silver/silver chloride/(0.1 M KCl) supplied by XTZ Ltd.

### Pretreatment of the Electrode

The working gold disk electrode was rinsed with deionized water and polished with 0.2 μm alumina (30 s) before and after every analysis. The working pencil electrode was rinsed with deionized water and sanded with a 800 Grit wet and dry sandpaper (30 s) before and after each analysis.

### Degassing

Unless stated otherwise, solutions were purged with nitrogen for a period of 10 min before every scan and coated with nitrogen during scans to prevent oxygen from re-entering the solution. All experiments were carried out at room temperature (15–18 °C).

## Results and Discussion

DNTs are known to be poorly soluble in aqueous media.^38^ Kong et al., however, reported that 2,4-DNT is more soluble in acidic solutions.^36^ 2,4-DNT was found to be more soluble in a solution of sulfuric acid where 200 ppm was dissolved as opposed to a 100 ppm limit typically reported for water. Sulfuric acid as a supporting electrolyte with a gold electrode serving as a working electrode was considered with reference to what was reported by McCormack et al.,^39^ where a reasonable potential window was observed below a potential of 800 mV.

**Figure 1:**
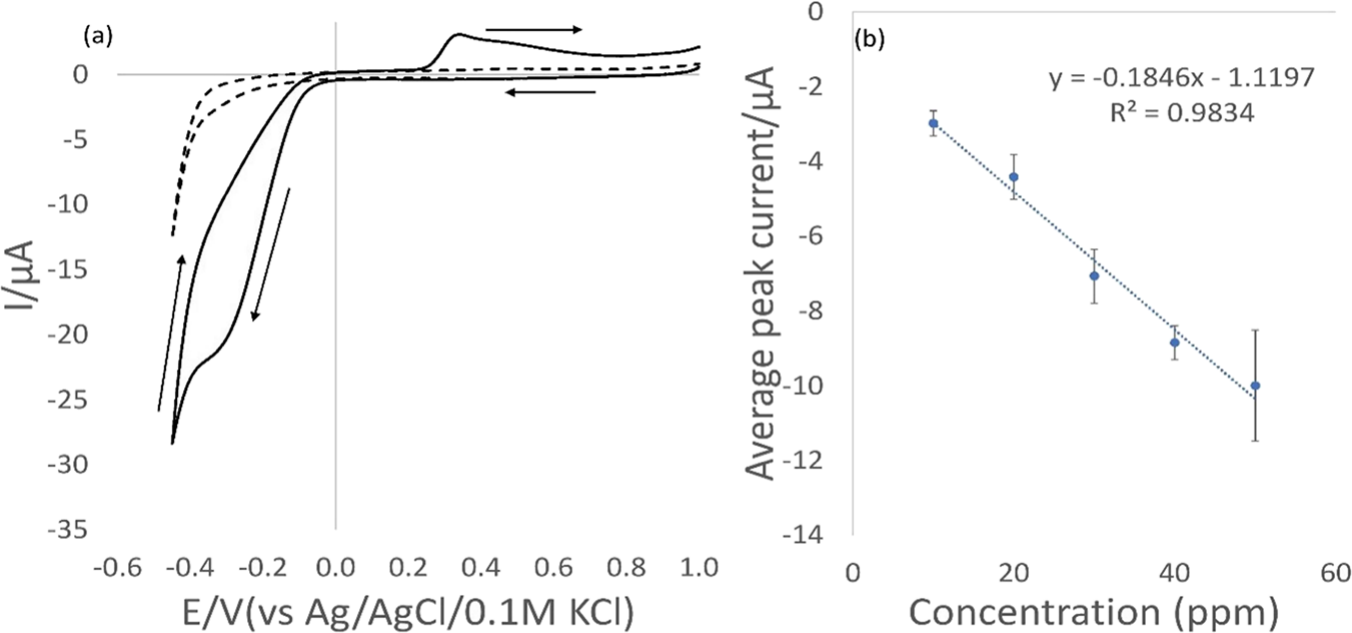
(a) CV scan of 2M H_2_SO_4_ (dashed line) and a scan of 100 ppm of DNT in 2M H_2_SO_4_ (solid line) at a scan rate of 50 mV/s using a gold electrode (aerated). (b) Plot of average peak current vs concentration in parts per million of DNT in 2M H_2_SO_4_ (*n* = 3).

On running a cathodic potential sweep with a gold electrode in 2M sulfuric acid as shown in Figure 1a, a clean potential window was observed between 1000 and −500 mV. At more negative potentials, HER as a result of interaction with the electrode occurs.^40^ At a concentration of 100 ppm, 2,4-DNT in the same electrolyte resulted in a cathodic sweep scan within the potential window, which is also shown in Figure 1a. A distinct peak at −300 mV in the forward cathodic and another peak at 400 mV on the anodic scan were observed, which is indicative of an irreversible electron transfer reaction.^19^ Neither of these peaks were observed in the background electrolyte solution of 2 M sulfuric acid, indicating that DNT is being detected in the CV scan.

Using peak current maxima for the calibration series, a reasonable linearity (*R*^2^ = 0.9834) and repeatability was observed (Figure 1b), based on a scan rate of 50 mV/s, without deaeration. Noteworthy was the fact that the cathodic peak potential was observed to be unstable around −300 mV between analysis. When deaeration of the background electrolyte of 2 M H_2_SO_4_ was introduced, the results shown in Figure S3 were observed. The background current for deaerated samples (oxygen removed) is absent at the relevant potential, compared to when deaeration is skipped (deaeration may not be an option in some analytical scenarios). This becomes relevant when concentrations lower than 4 ppm are being analyzed. Further experiments were conducted under deaerated conditions to focus on the detection limits of the system.

**Figure 2:**
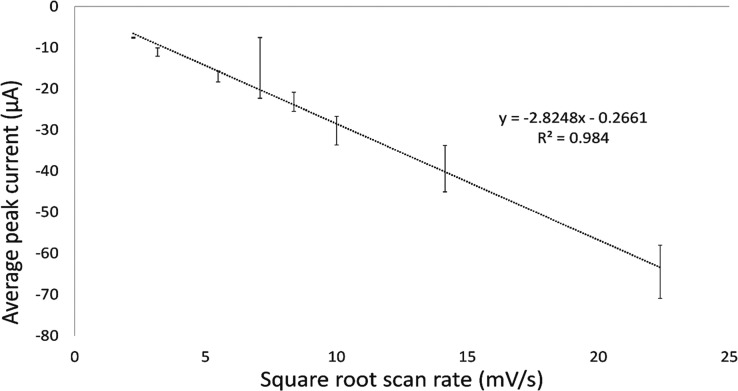
Linear plot obtained of the average peak current vs the square root of scan rate (*n* = 5).

**Figure 3:**
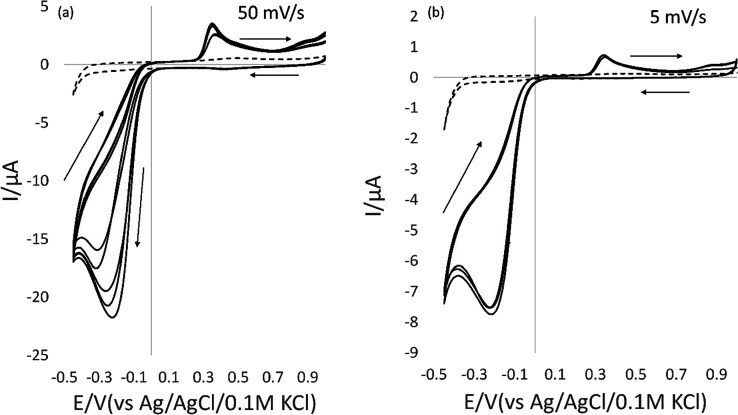
Multiple scans of 100 ppm of 2,4-DNT in 2 M H_2_SO_4_ at scan rates of (a) 50 mV/s and (b) 5 mV/s (*n* = 5).

The effect of the scan rate was investigated next. In Figure 2, the general trend of improving repeatability when moving from fast to slow scan rates is shown. More specifically, in Figure 3, the effect of changing from fast (50 mV/s) to slow (5 mV/s) scan rates shows better repeatability. Also of note is that the reduction voltage peak position becomes more stable at 5 mV/s. This finding is further supported by the work of Mbah et al. which suggested that slower sweep rates were more effective for the complete oxidation of DNT, which was reported in experiments with fast sweep rates above 400 mV/s.^41^

A linear plot obtained between the average peak current (*n* = 5) versus the square root of the scan rate suggests a diffusion-controlled mechanism which is again supported by the work of Mbah et al.^41^ All further work was carried out at 5 mV/s to optimize repeatability.

**Figure 4:**
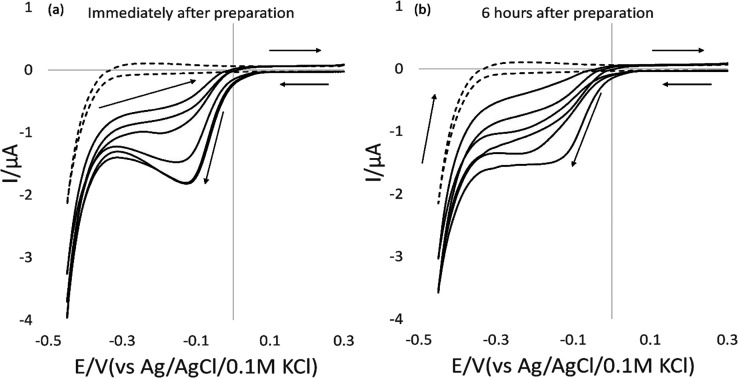
CV scans of 20 ppm of 2,4 DNT in 2 M H_2_SO_4_ at a scan rate of 5 mV/s (a) immediately after preparation and (b) after 6 h of preparation (*n* = 3).

The use of sulfuric acid is well established for the manufacture of nitroaromatics as it is known to improve aqueous solubility, but it is also acknowledged that oxidation and hydrolysis can be observed over time.^42^ Thus, the stability of the analyte solution was considered immediately after preparation and within the first 6 h (Figure 4). It was found that the repeatability and stability of the 2,4-DNT solution was affected after 6 h such that the % RSD increased from 11% for analysis immediately after sample preparation for 20 ppm of 2,4 DNT to 23.6% for the same solution after 6 h. It was also evident that the potentials at which the reduction peak appeared differed with every scan after 6 h of preparation. The kinetic lability of 2,4-DNT is thus further highlighted by these time-dependent comparisons. We therefore recommend that analysis is completed within 3 h of sample preparation.

**Figure 5:**
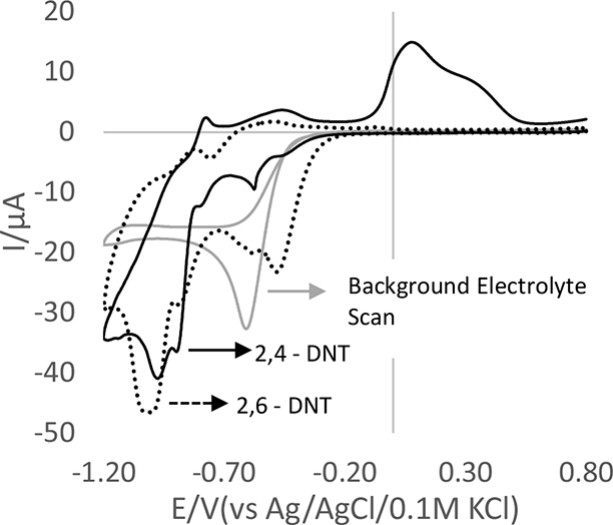
CV comparison of 100 ppm of 2,4-DNT and 2,6-DNT in acetonitrile in 100 mM sodium perchlorate at 50 mV/s using a gold electrode.

Only one peak associated with DNT was observed in the cathodic sweep in the H_2_SO_4_ system which is different from additional peaks reported in other systems.^43,44^ Experiments were conducted in acetonitrile with the added 0.1 M sodium perchlorate supporting electrolyte, 100 ppm of 2,4-DNT, and 2,6-DNT separately. Figure 5 reveals separate reduction peaks associated with the two nitro groups of 2,4-DNT and 2,6-DNT. The peak associated with the 2-nitro group (−0.88 V) was similar in both compounds, whereas the peaks associated with the 4- (−0.96 V) and 6-nitro groups (−1.0 V) were considerably different, with the 6-nitro group being broader than the 4-nitro group. A similarly broad 6-nitro peak can also be seen in the work reported by Toh et al., indicating the possibility of differentiating between 2,4- and 2,6-DNT.^13^

**Figure 6:**
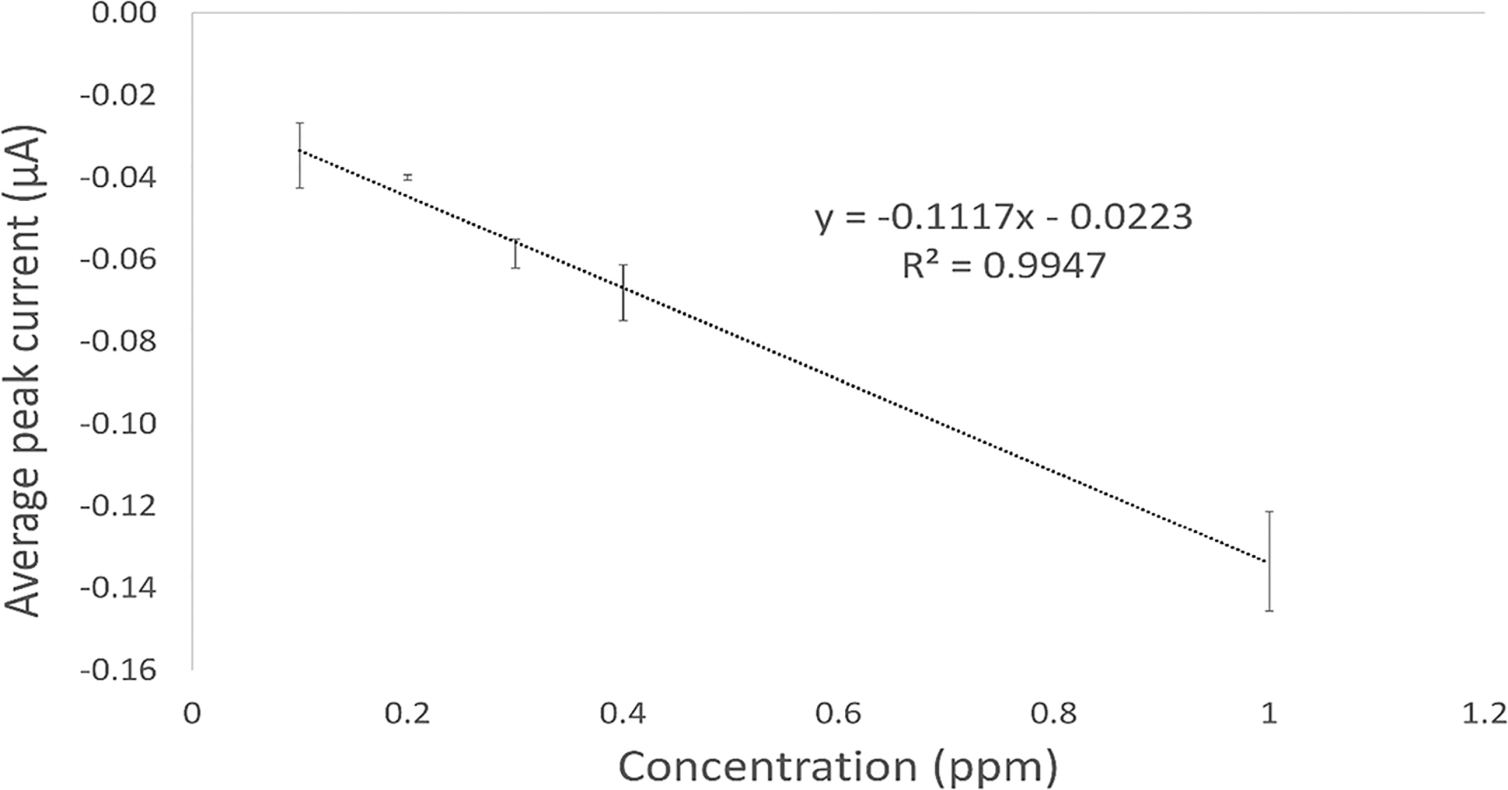
Calibration plot of 2,4-DNT at a scan rate of 5 mV/s in the degassed range 0.1–1.0 ppm (*n* = 3).

The determination of the limit of detection (LOD) and limit of quantification (LOQ) of the gold working electrode system was also performed. The calibration plot (Figure 6) is linear in the low concentration range of 0.1–1 ppm with the regression equation: −*Ip* (peak current) = −0.1117 *C* – 0.0223, where *C* is concentration. This has a correlation coefficient (*R*^2^) of 0.9947. In the present investigation, the LOD and LOQ were based on 3 × s/m and 10 × s/m, respectively, where s is the standard deviation of the peak currents (n = 3, three runs) and m is the slope of the calibration plot.^45^

The LOD was estimated to be 0.158 ppm (0.87 μM) and 0.480 ppm (2.64 μM) was found to be the LOQ. The sensitivity was determined as 0.1117 μA/μM. This indicated that 2,4-DNT can be determined in the given concentration range (0.1–1 ppm) using this gold electrode/H_2_SO_4_ system.

**Figure 7:**
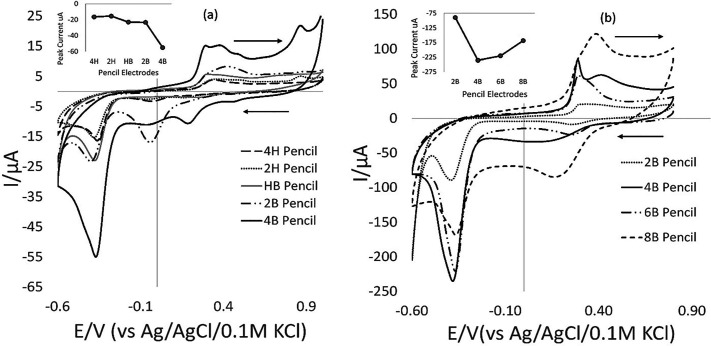
(a) 50 mV/s CV scans of 100 ppm 2,4-DNT comparing different pencils: 4H, 2H, HB, 2B, and 4B (inset) and plots of peak current vs pencil electrodes. (b) 50 mV/s CV scans of 100 ppm 2,4-DNT comparing different B-grade pencils 2B, 4B, 6B, and 8B (inset) and plots of peak current vs pencil electrodes.

The gold electrode used in this work and the various modified electrodes used in other literature can be considered as expensive electrodes; thus, a cheaper widely accessible alternative was considered in the form of pencil electrodes. Commercially available pencils from the Faber Castell 9000 series (standard wood-based stationery pencils) were tested after a brief sanding of the distal flat end. Pencil types 4H, 2H, HB, 2B, 4B, 6B, and 8B were examined. A general observation was that B pencils produced a larger current than HB pencils, which in turn produced greater currents than H pencils. It is known that higher B numbers represent a higher graphite content (graphite is a well-known electroactive material^46^) relative to clay (the other major component of pencil “leads”). It is not simply a case of using the softest (highest graphite content) pencil available in a series such as 8B, as the signal-to-noise ratio or peak current quality may suffer.^47^ Experimental determination is therefore essential. The 4B pencil was found to be the most suitable in our case based on the optimum peak current and shape (Figure 7). It is also noticeable that higher B number pencils are physically softer and more brittle in nature and therefore are more prone to breaking during use. Robustness considerations are all the more important for field-use instruments where operators may have received minimal training.

In contrast to gold, the pencil electrodes we examined were found to be adsorption-controlled, as a linear plot was obtained between the peak current and scan rate which was not obtained when plotted against the square root of the scan rate (Supporting Information Figure S2). The graphite carbon electrode material likely offers an ideal van der Waals substrate for binding small organic molecules such as nitrotoluenes via dispersion forces, explaining the observed adsorption control observed. Repeat scans on this electrode (without surface cleaning between runs) resulted in a diminishing peak indicating the effect of adsorption (Supporting Information Figure S1) and an increasingly blocked electrode surface; thus, the surface of the 4B pencil electrode was simply polished with a 800 grade sandpaper between each run for a fresh surface. The polishing of the WE surface effectively represents using a different pencil lead each time and gives an indication of reproducibility when using different pencil units from the same commercial series. This also mimics a single-use electrode scenario, which may eventually be preferred for a field-deployable system or where only the simplest electrode-polishing regime (brief polishing with a sandpaper) is tolerable. A 9.2% RSD at a scan rate of 5 mV/s (*n* = 3) was thus observed for a 5 ppm 2,4-DNT solution. Each analysis was done in triplicate to examine precision. A scan rate study (Supporting Information Figure S2) using pencil electrodes did not show much difference in repeatability between the slower scan rates and was significantly better than that observed at faster scan rates.

**Figure 8:**
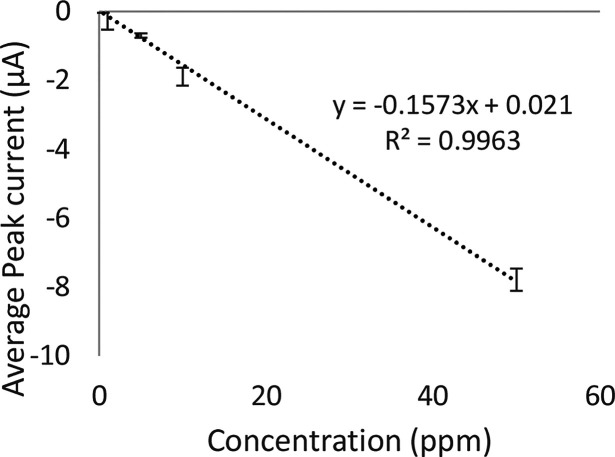
Calibration plot of 2,4-DNT using a 4B pencil electrode at a scan rate of 5 mV/s in the degassed range 1–50 ppm (*n* = 3).

The calibration plot is linear in the concentration range of 1–50 ppm (Figure 8) with the regression equation: −*Ip* = −0.1573 *C* + 0.021. This has a correlation coefficient (*R*^2^) of 0.9963. The LOD was estimated to be 4.8 ppm (26.48 μM), and 14.6 ppm (80.54 μM) was found to be the LOQ. The sensitivity was determined as 0.1573 μA/μM.

The observed single peak in H_2_SO_4_ systems was true for 2,4-DNT (as seen in Figure S4) using both gold and pencil working electrodes, which suggests either the merging of two reduction events into a single peak in protic media as was reported in the work of Buhlmann et al.^48^ or only the reduction of the 2-nitro group observed at acidic pH. Apart from the mechanistic insight, the observed single peak suffices for analytical purposes.

Table 1 compares the two-electrode systems discussed in this paper with other electrodes in the literature in terms of 2,4-DNT analysis, revealing comparable LODs. Central to our work was keeping the electrode material, electrolyte, and electrochemical mode (CV) as simple and accessible as possible. This can be advantageous for the further development of simple and field-deployable electrochemical systems, for example, in remote locations with contaminated soils or water, where the complexity of sample processing, analysis, and operator training have to be kept as uncomplicated as possible.

**Table 1:** Comparison of the Literature Methods for Electrochemical 2,4-DNT Analysis^a^

electrode	technique	LOD	LOQ	ref
gold nanoparticles/poly(carbazole-aniline) film-modified glassy carbon sensor electrodes	SWV	30 μM	100 μM	(12)
Fe-doped inorganic	DPV	4.64 nM		(11)
“o-CoxFe1-xSe2 solid”				
graphene obtained in LiClO_4_	DPV	2.73 ppm (15.03 μM)		(49)
SPCE	CV	0.7 μM		(10)
silver-modified carbon fiber electrode	CV	5 μM		(41)
three-dimensionally ordered macroporous carbon electrode	SWV	10 μM		(9)
bare gold electrode	CV	0.158 ppm (0.87 μM)	0.48 ppm (2.64 μM)	this work
4B pencil Faber Castell	CV	4.8 ppm (26.48 μM)	14.6 ppm (80.54 μM)	this work

a CV = cyclic voltammetry. SWV = square-wave voltammetry. DPV = differential pulse voltammetry.

As seen in Table 1, the LODs and LOQs obtained from the gold and pencil electrodes (present work) have been compared with relevant literature work (limited work available from the last 20 years). In general, works to date rely on specialist modified electrode materials, as well as carefully buffered samples/electrolytes. Conversely, we combine affordable and ubiquitous electrodes (pencil) with a simple one-component electrolyte (common acid). In combination with the most simple of electrochemical experiments (CV), our systems already demonstrates comparable LODs and LOQs to those reported in the literature (Table 1). Our gold electrode method is found to have a lower LOD than most others reported at 0.87 μM, while the LOD of our proposed pencil electrode is higher at 26 μM.

## Optimizations

The experiment underwent optimization by adjusting multiple parameters. To enhance the analysis at lower concentrations, the solution underwent deaeration. The scan rate was reduced to 5 mV/s to enable completion of the reaction mechanism, and this improved the repeatability of the experiment when using gold electrodes. Sample optimization revealed that the best analysis occurred within 6 h of preparation. Multiple pencil electrodes, spanning from 4H to 8B, were experimented with DNT, ultimately pinpointing that the 4B pencil electrode yielded the most optimal results.

## Conclusions

The solubility of 2,4-DNT was found to increase with the use of 2 M H_2_SO_4_ as the electrolyte. In water alone, we typically observed a DNT solubility limit of 100 ppm compared to at least 300 ppm in 2 M H_2_SO_4–._ Distinct peaks were obtained on using CV, which increased linearly with the concentration of 2,4-DNT using both gold electrodes and 4B Faber Castell pencil electrodes. It was found that analysis could be conducted at higher concentrations of DNT, up to 4 ppm, without deaeration but was found to be essential for precise measurement at lower concentrations. In some field testing locations, such knowledge may be pertinent. The mechanism behind the DNT redox reaction was found to be diffusion-controlled for gold WEs and adsorption-controlled for pencil-based electrodes. The diffusion-controlled nature confirmed for our gold electrodes is the likely reason why repeatability improves for slow (5 mV/s) scan rates, thus providing enough time for a complete and repeatable DNT reduction to be observed.

Analysis within 6 h of sample preparation was deemed necessary, as variance was observed after this, pointing to the labile nature of 2,4-DNT in acid medium. In organic media, two peaks were observed for both 2,4-DNT and 2,6-DNT, with the peaks associated with the 4- and 6-nitro groups being visibly distinct. The scans of 2,4-DNT and 2,6-DNT in H_2_SO_4_ were carried out with both gold and pencil electrodes, with voltammograms possibly merging to a single peak or only one nitro group reduction being apparent. Future investigations will clarify this. Although 2,4- and 2,6-DNT could no longer be distinguished, effective quantification still takes place. CV analysis with gold electrodes produced LODs estimated to be 0.158 ppm (0.87 μM), and 0.480 ppm (2.64 μM) was found to be the LOQ. With a pencil electrode, the LOD was estimated to be 4.8 ppm (26.3 μM), and 14.6 ppm (80.52 μM) was found to be the LOQ.

Future work includes improving the detection limits and sensitivity for the pencil electrodes, for example, through the use of sensor arrays and multiplexing. Other pencil electrode geometries will be investigated, entailing miniaturization and custom-built shapes (not restricted to commercially available stationery samples). Chemical etching, for example, by liquid-phase exfoliation, hot nitric acid treatment, or oxygen plasma exposure is known to introduce mesoporous features on the graphite-based electrode and increase the surface area (hence electrode signal). An improved and consistent electrode surface area may be achievable by chemical rather than physical (manual sanding/abrasion) means, enhancing the electrode signal and improving reproducibility. The combination of pencil leads, H_2_SO_4_ electrolyte, and CV remains the basis for a simple, field-deployable, and inexpensive method of DNT detection.

## Supporting Information Available

Consecutive scans of 2,4-DNT at 50 mV/s scan rate using a 4B pencil electrode; plot between the average peak current vs scan rate and the square root of the scan rate of 2,4-DNT using a 4B pencil electrode; CV scans of de-aerated 2 M H_2_SO_4_ and non-de-aerated 2 M H_2_SO_4_ at a scan rate of 50 mV/s; CV scans of background electrolyte, 100 ppm 2,4 DNT, at 50 mV/s using a gold electrode and a 4B Faber Castell pencil electrode; CV scan of 10 to 50 ppm DNT in 2 M H_2_SO_4_ at a scan rate of 50 mV/s using a gold electrode (aerated) and a plot of anodic average peak current vs concentration in ppm of DNT in 2 M H_2_SO_4_ (*n* = 3); and linear plot obtained of average *anodic* peak current vs square root of scan rate (*n* = 3) (PDF)

## Abbreviations

DNT: dinitrotoluene
WE: working electrode
CV: cyclic voltammetry
LOD: limit of detection
LOQ: limit of quantification
EPA: environmental protection agency
RSD: relative standard deviation
PPM: parts per million
SWV: square wave voltammetry
DPV: differential pulse voltammetry
HER: hydrogen evolution reaction

## References

[ref1] Xiao H.; Liu R.; Zhao X.; Qu J. Enhanced Degradation of 2,4-Dinitrotoluene by Ozonation in the Presence of Manganese(II) and Oxalic Acid. J. Mol. Catal. A Chem. 2008, 286 (1–2), 149–155. 10.1016/j.molcata.2008.02.013.

[ref2] Dargahi A.; Vosoughi M.; Ahmad Mokhtari S.; Vaziri Y.; Alighadri M. Electrochemical Degradation of 2,4-Dinitrotoluene (DNT) from Aqueous Solutions Using Three-Dimensional Electrocatalytic Reactor (3DER): Degradation Pathway, Evaluation of Toxicity and Optimization Using RSM-CCD. Arabian Journal of Chemistry 2022, 15 (3), 103648 10.1016/j.arabjc.2021.103648.

[ref3] Lent E. M. Wildlife Toxicity Assessment for 2,4-Dinitrotoluene and 2,6-Dinitrotoluene. In Wildlife Toxicity Assessments for Chemicals of Military Concern; Elsevier, 2015; pp 107–146.

[ref4] Agency for Toxic Substances and Disease Registry (US). Toxicological Profile for Dinitrotoluenes, 2016. 37184178

[ref5] https://aoav.org.uk/2020/explosive-violence-in-2019/. Deaths and Injuries Caused by Explosives in 2019.

[ref6] Young R. A. Dinitrotoluene. In Encyclopedia of Toxicology; Elsevier, 2005; pp 60–63.

[ref7] Caygill J. S.; Davis F.; Higson S. P. J. Current Trends in Explosive Detection Techniques. Talanta 2012, 88, 14–29. 10.1016/j.talanta.2011.11.043. 22265465

[ref8] Ahmadi S. A.; Tajik S. Efficient Detection of Droxidopa in the Presence of Carbidopa Using a Modified Screen-Printed Graphite Electrode. Top. Catal. 2023, 10.1007/s11244-023-01866-9.

[ref9] Fierke M. A.; Olson E. J.; Bühlmann P.; Stein A. Receptor-Based Detection of 2,4-Dinitrotoluene Using Modified Three-Dimensionally Ordered Macroporous Carbon Electrodes. ACS Appl. Mater. Interfaces 2012, 4 (9), 4731–4739. 10.1021/am301108a. 22905948

[ref10] Caygill J. S.; Collyer S. D.; Holmes J. L.; Davis F.; Higson S. P. J. Disposable Screen-Printed Sensors for the Electrochemical Detection of TNT and DNT. Analyst 2013, 138 (1), 346–352. 10.1039/C2AN36351H. 23152954

[ref11] Xia X.; Liu Z.; Xu Q.-Q.; Cheng X.-L.; Li J.-J.; Li S.-S. Ultra-Sensitive Electroanalysis of Toxic 2,4-DNT on o-CoxFe1-XSe2 Solid Solution: Fe-Doping-Induced c-CoSe2 Phase Transition to Form Electron-Rich Active Sites. Anal. Chim. Acta 2022, 1227, 340291 10.1016/j.aca.2022.340291. 36089310

[ref12] Sağlam Ş.; Üzer A.; Erçağ E.; Apak R. Electrochemical Determination of TNT, DNT, RDX, and HMX with Gold Nanoparticles/Poly(Carbazole-Aniline) Film–Modified Glassy Carbon Sensor Electrodes Imprinted for Molecular Recognition of Nitroaromatics and Nitramines. Anal. Chem. 2018, 90 (12), 7364–7370. 10.1021/acs.analchem.8b00715. 29786423

[ref13] Toh H. S.; Ambrosi A.; Pumera M. Electrocatalytic Effect of ZnO Nanoparticles on Reduction of Nitroaromatic Compounds. Catal. Sci. Technol. 2013, 3 (1), 123–127. 10.1039/C2CY20253K.

[ref14] Zhang Z.; Karimi-Maleh H. Label-Free Electrochemical Aptasensor Based on Gold Nanoparticles/Titanium Carbide MXene for Lead Detection with Its Reduction Peak as Index Signal. Adv. Compos Hybrid Mater. 2023, 6 (2), 68 10.1007/s42114-023-00652-1.

[ref15] Karimi-Maleh H.; Liu Y.; Li Z.; Darabi R.; Orooji Y.; Karaman C.; Karimi F.; Baghayeri M.; Rouhi J.; Fu L.; Rostamnia S.; Rajendran S.; Sanati A. L.; Sadeghifar H.; Ghalkhani M. Calf Thymus Ds-DNA Intercalation with Pendimethalin Herbicide at the Surface of ZIF-8/Co/RGO/C3N4/Ds-DNA/SPCE; A Bio-Sensing Approach for Pendimethalin Quantification Confirmed by Molecular Docking Study. Chemosphere 2023, 332, 138815 10.1016/j.chemosphere.2023.138815. 37146774

[ref16] Karimi F.; Karimi-Maleh H.; Rouhi J.; Zare N.; Karaman C.; Baghayeri M.; Fu L.; Rostamnia S.; Dragoi E. N.; Ayati A.; Krivoshapkin P. Revolutionizing Cancer Monitoring with Carbon-Based Electrochemical Biosensors. Environ. Res. 2023, 239, 117368 10.1016/j.envres.2023.117368. 37827366

[ref17] Karimi-Maleh H.; Darabi R.; Baghayeri M.; Karimi F.; Fu L.; Rouhi J.; Niculina D. E.; Gündüz E. S.; Dragoi E. N. Recent Developments in Carbon Nanomaterials-Based Electrochemical Sensors for Methyl Parathion Detection. Journal of Food Measurement and Characterization 2023, 17 (5), 5371–5389. 10.1007/s11694-023-02050-z.

[ref18] Zhang Z.; Karimi-Maleh H. In Situ Synthesis of Label-Free Electrochemical Aptasensor-Based Sandwich-like AuNPs/PPy/Ti3C2Tx for Ultrasensitive Detection of Lead Ions as Hazardous Pollutants in Environmental Fluids. Chemosphere 2023, 324, 138302 10.1016/j.chemosphere.2023.138302. 36871797

[ref19] Bard A. J.; Faulkner L. R. Electrochemical Methods: Fundamentals and Applications, 2nd ed.; Wiley, 2001.

[ref20] Elgrishi N.; Rountree K. J.; McCarthy B. D.; Rountree E. S.; Eisenhart T. T.; Dempsey J. L. A Practical Beginner’s Guide to Cyclic Voltammetry. J. Chem. Educ. 2018, 95 (2), 197–206. 10.1021/acs.jchemed.7b00361.

[ref21] Shashanka R.; Kumara Swamy B. E. Simultaneous Electro-Generation and Electro-Deposition of Copper Oxide Nanoparticles on Glassy Carbon Electrode and Its Sensor Application. SN Appl. Sci. 2020, 2 (5), 956 10.1007/s42452-020-2785-1.

[ref22] Rajendrachari S.; Basavegowda N.; Adimule V. M.; Avar B.; Somu P.; R M.; S K.; Baek K.-H. Assessing the Food Quality Using Carbon Nanomaterial Based Electrodes by Voltammetric Techniques. Biosensors 2022, 12 (12), 1173 10.3390/bios12121173. 36551140 PMC9775119

[ref23] Rajendrachari S.; Adimule V.; Gulen M.; Khosravi F.; Somashekharappa K. K. Synthesis and Characterization of High Entropy Alloy 23Fe-21Cr-18Ni-20Ti-18Mn for Electrochemical Sensor Applications. Materials 2022, 15 (21), 7591 10.3390/ma15217591. 36363181 PMC9657540

[ref24] Annu; Sharma S.; Jain R.; Raja A. N. Review—Pencil Graphite Electrode: An Emerging Sensing Material. J. Electrochem. Soc. 2020, 167 (3), 037501 10.1149/2.0012003JES.

[ref25] Trucano P.; Chen R. Structure of Graphite by Neutron Diffraction. Nature 1975, 258 (5531), 136–137. 10.1038/258136a0.

[ref26] Kung C.-T.; Hou C.-Y.; Wang Y.-N.; Fu L.-M. Microfluidic Paper-Based Analytical Devices for Environmental Analysis of Soil, Air, Ecology and River Water. Sens Actuators B Chem. 2019, 301, 126855 10.1016/j.snb.2019.126855.

[ref27] Trachioti M. G.; Hemzal D.; Hrbac J.; Prodromidis M. I. Generation of Graphite Nanomaterials from Pencil Leads with the Aid of a 3D Positioning Sparking Device: Application to the Voltammetric Determination of Nitroaromatic Explosives. Sens Actuators B Chem. 2020, 310, 127871 10.1016/j.snb.2020.127871.

[ref28] Ryan P.; Zabetakis D.; Stenger D.; Trammell S. Integrating Paper Chromatography with Electrochemical Detection for the Trace Analysis of TNT in Soil. Sensors 2015, 15 (7), 17048–17056. 10.3390/s150717048. 26184223 PMC4541921

[ref29] Baysal G.; Uzun D.; Hasdemir E. The Fabrication of a New Modified Pencil Graphite Electrode for the Electrocatalytic Reduction of 2-Nitrophenol in Water Samples. J. Electroanal. Chem. 2020, 860, 113893 10.1016/j.jelechem.2020.113893.

[ref30] Asadpour-Zeynali K.; Najafi-Marandi P. Bismuth Modified Disposable Pencil-Lead Electrode for Simultaneous Determination of 2-Nitrophenol and 4-Nitrophenol by Net Analyte Signal Standard Addition Method. Electroanalysis 2011, 23 (9), 2241–2247. 10.1002/elan.201100103.

[ref31] Sree V. G.; Sohn J. I.; Im H. Pre-Anodized Graphite Pencil Electrode Coated with a Poly(Thionine) Film for Simultaneous Sensing of 3-Nitrophenol and 4-Nitrophenol in Environmental Water Samples. Sensors 2022, 22 (3), 1151 10.3390/s22031151. 35161895 PMC8838205

[ref32] Nie D.; Li P.; Zhang D.; Zhou T.; Liang Y.; Shi G. Simultaneous Determination of Nitroaromatic Compounds in Water Using Capillary Electrophoresis with Amperometric Detection on an Electrode Modified with a Mesoporous Nano-Structured Carbon Material. Electrophoresis 2010, 31 (17), 2981–2988. 10.1002/elps.201000275. 20836147

[ref33] Karikalan N.; Kubendhiran S.; Chen S.-M.; Sundaresan P.; Karthik R. Electrocatalytic Reduction of Nitroaromatic Compounds by Activated Graphite Sheets in the Presence of Atmospheric Oxygen Molecules. J. Catal. 2017, 356, 43–52. 10.1016/j.jcat.2017.09.012.

[ref34] Gupta R.; Rastogi P. K.; Ganesan V.; Yadav D. K.; Sonkar P. K. Gold Nanoparticles Decorated Mesoporous Silica Microspheres: A Proficient Electrochemical Sensing Scaffold for Hydrazine and Nitrobenzene. Sens Actuators B Chem. 2017, 239, 970–978. 10.1016/j.snb.2016.08.117.

[ref35] Trachioti M. G.; Hemzal D.; Hrbac J.; Prodromidis M. I. Generation of Graphite Nanomaterials from Pencil Leads with the Aid of a 3D Positioning Sparking Device: Application to the Voltammetric Determination of Nitroaromatic Explosives. Sens Actuators B Chem. 2020, 310, 127871 10.1016/j.snb.2020.127871.

[ref36] Kong L. Q.; Li Y. G.; Zheng S. Q. Measurement and Correlation of Solubility for 2,4-Dinitrotoluent in Mixed Solvents of Water and Nitric Acid. Adv. Mat Res. 2011, 233–235, 1332–1335. 10.4028/www.scientific.net/AMR.233-235.1332.

[ref37] Song K.; Meng Q.; Shu F.; Ye Z. Recovery of High Purity Sulfuric Acid from the Waste Acid in Toluene Nitration Process by Rectification. Chemosphere 2013, 90 (4), 1558–1562. 10.1016/j.chemosphere.2012.09.043. 23047120

[ref38] Phelan J. M.; Barnett J. L. Solubility of 2,4-Dinitrotoluene and 2,4,6-Trinitrotoluene in Water. J. Chem. Eng. Data 2001, 46 (2), 375–376. 10.1021/je000300w.

[ref39] McCormick W.; McDonagh P.; Doran J.; McCrudden D. Covalent Immobilisation of a Nanoporous Platinum Film onto a Gold Screen-Printed Electrode for Highly Stable and Selective Non-Enzymatic Glucose Sensing. Catalysts 2021, 11 (10), 1161 10.3390/catal11101161.

[ref40] Xu X.; Makaraviciute A.; Pettersson J.; Zhang S.-L.; Nyholm L.; Zhang Z. Revisiting the Factors Influencing Gold Electrodes Prepared Using Cyclic Voltammetry. Sens Actuators B Chem. 2019, 283, 146–153. 10.1016/j.snb.2018.12.008.

[ref41] Mbah J.; Moorer K.; Pacheco-Londoño L.; Hernandez-Rivera S.; Cruz G. Zero Valent Silver-Based Electrode for Detection of 2,4,-Dinitrotoluene in Aqueous Media. Electrochim. Acta 2013, 88, 832–838. 10.1016/j.electacta.2012.10.068.

[ref42] Calvo R.; Zhang K.; Passera A.; Katayev D. Facile Access to Nitroarenes and Nitroheteroarenes Using N-Nitrosaccharin. Nat. Commun. 2019, 10 (1), 3410 10.1038/s41467-019-11419-y. 31363083 PMC6667458

[ref43] Xia X.; Liu Z.; Xu Q.-Q.; Cheng X.-L.; Li J.-J.; Li S.-S. Ultra-Sensitive Electroanalysis of Toxic 2,4-DNT on o-CoxFe1-XSe2 Solid Solution: Fe-Doping-Induced c-CoSe2 Phase Transition to Form Electron-Rich Active Sites. Anal. Chim. Acta 2022, 1227, 340291 10.1016/j.aca.2022.340291. 36089310

[ref44] Yew Y. T.; Ambrosi A.; Pumera M. Nitroaromatic Explosives Detection Using Electrochemically Exfoliated Graphene. Sci. Rep 2016, 6 (1), 33276 10.1038/srep33276. 27633489 PMC5025880

[ref45] Yilmaz B.; Kaban S.; Akcay B. K.; Ciltas U. Differential Pulse Voltammetric Determination of Diclofenac in Pharmaceutical Preparations and Human Serum. Brazilian Journal of Pharmaceutical Sciences 2015, 51 (2), 285–294. 10.1590/S1984-82502015000200005. PMC4518099 26330859

[ref46] Greenwood N. N.; Earnshaw A. Chemistry of the Elements, 2nd ed.; Elsevier, 2012.

[ref47] Torrinha Á.; Amorim C. G.; Montenegro M. C. B. S. M.; Araújo A. N. Biosensing Based on Pencil Graphite Electrodes. Talanta 2018, 190, 235–247. 10.1016/j.talanta.2018.07.086. 30172505

[ref48] Olson E. J.; Isley W. C.; Brennan J. E.; Cramer C. J.; Bühlmann P. Electrochemical Reduction of 2,4-Dinitrotoluene in Aprotic and PH-Buffered Media. J. Phys. Chem. C 2015, 119 (23), 13088–13097. 10.1021/acs.jpcc.5b02840.

[ref49] Yew Y. T.; Ambrosi A.; Pumera M. Nitroaromatic Explosives Detection Using Electrochemically Exfoliated Graphene. Sci. Rep 2016, 6 (1), 33276 10.1038/srep33276. 27633489 PMC5025880

