# Supplementary material for: Pencil and Gold Electrode Materials for the Electrochemical Study and Analysis of Dinitrotoluene
Source: ACS Omega. 2024 Feb 12;9(8):9375–82. doi: 10.1021/acsomega.3c08741 (PMC10905693; doi:10.1021/acsomega.3c08741)
Supplement: Supplementary file 1 — ao3c08741_si_001.pdf [file ao3c08741_si_001.pdf]

Supplementary Information for Pencil and gold electrode materials for the electrochemical study and analysis of Dinitrotoluenes.

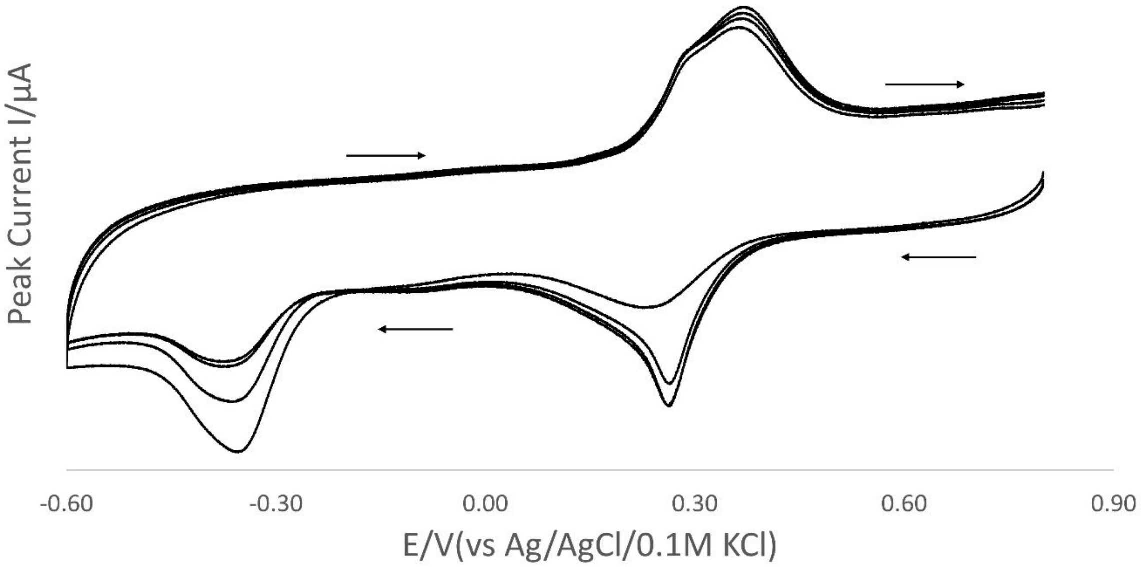

Figure S1. Consecutive repeat cyclic voltammetry at scan rate 50mV/s using 4B pencil electrode of 100 [illegible] 2,4- DNT

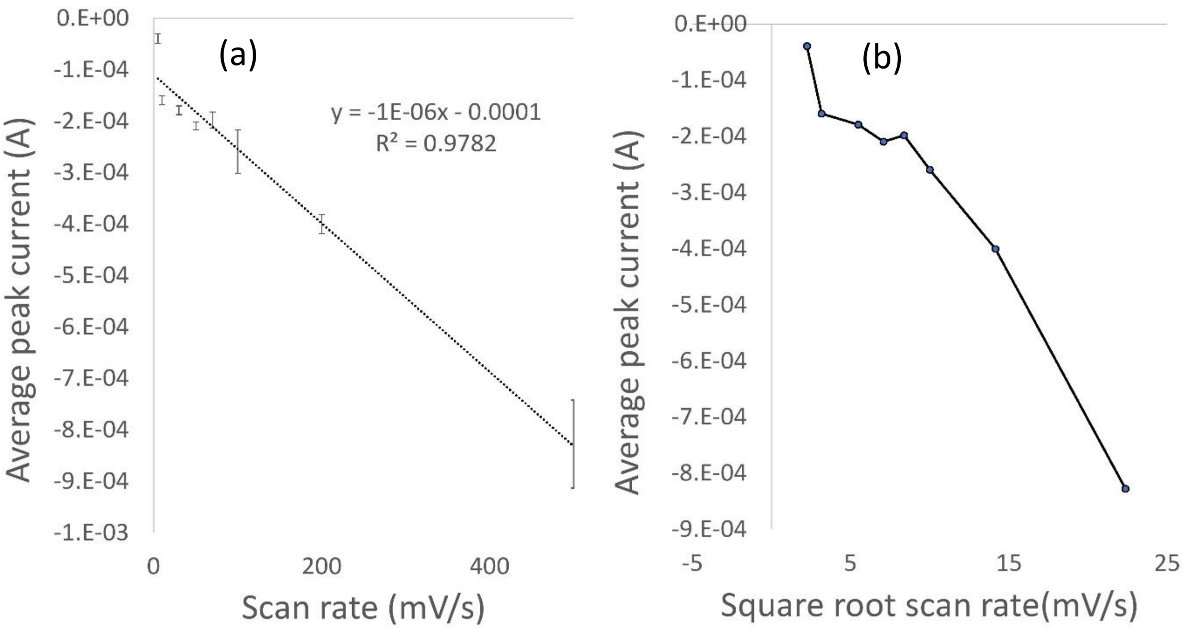

Figure S2. (a) Plot between average peak current vs scan rate of 4B pencil electrode. (b) Plot between average peak current vs square root of scan rate of 4B pencil electrode.

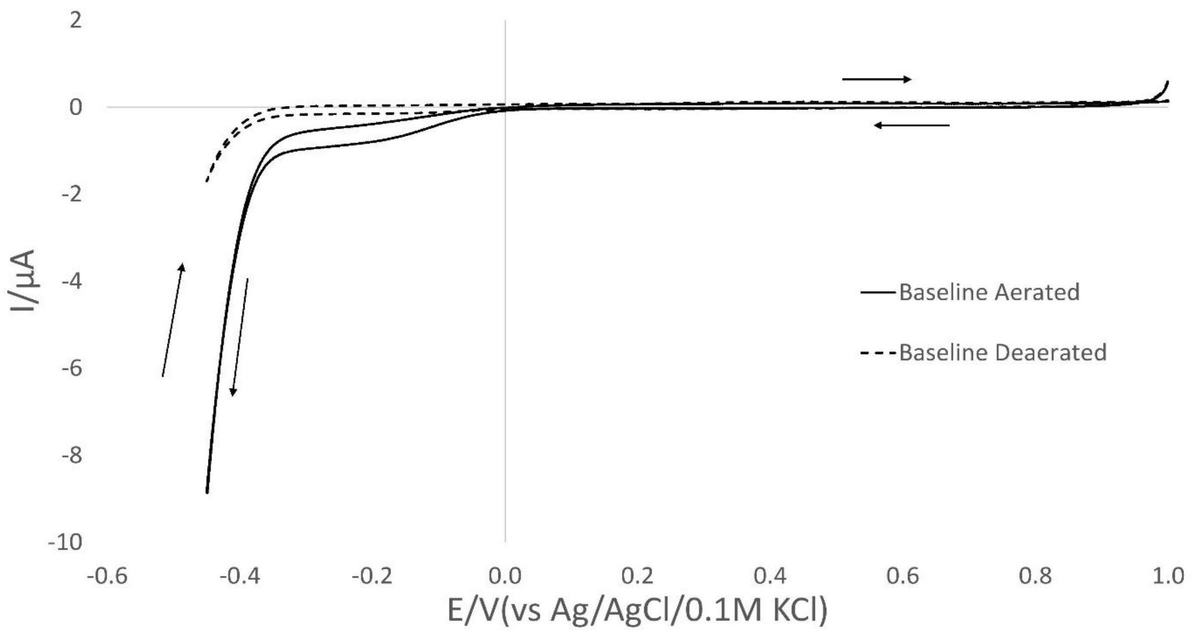

Figure S3. CV Scans of deaerated 2 M H_2SO_4 (dashed) and non-deaerated 2 M H_2SO_4 (solid) at scan rate of 50 mV/s.

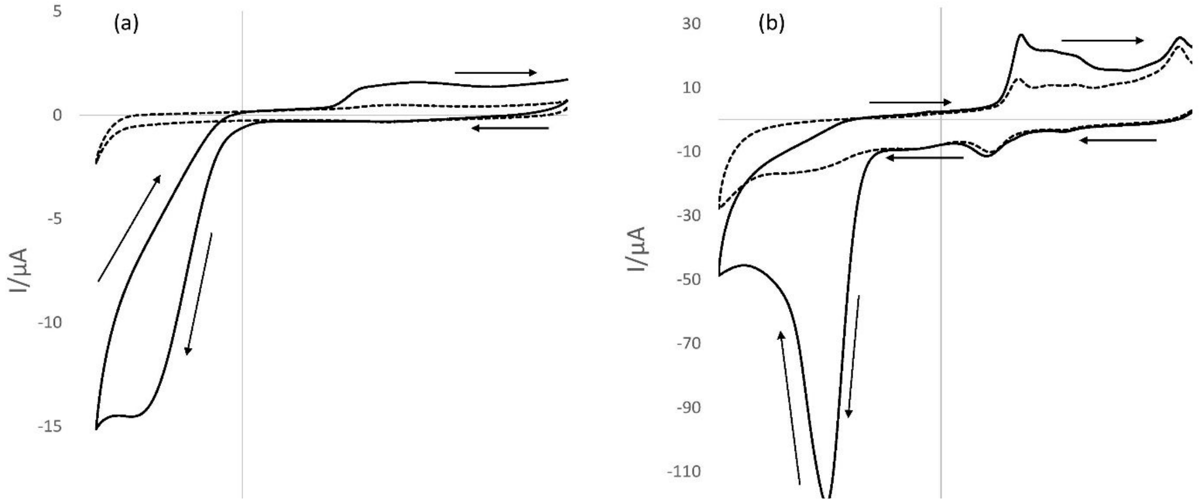

Figure S4. CV Scans of Background electrolyte (Dotted line), 100 ppm 2,4 DNT (Solid line) at 50 mV/s using (a) Gold electrode (b) 4B FaberCastel pencil electrode.

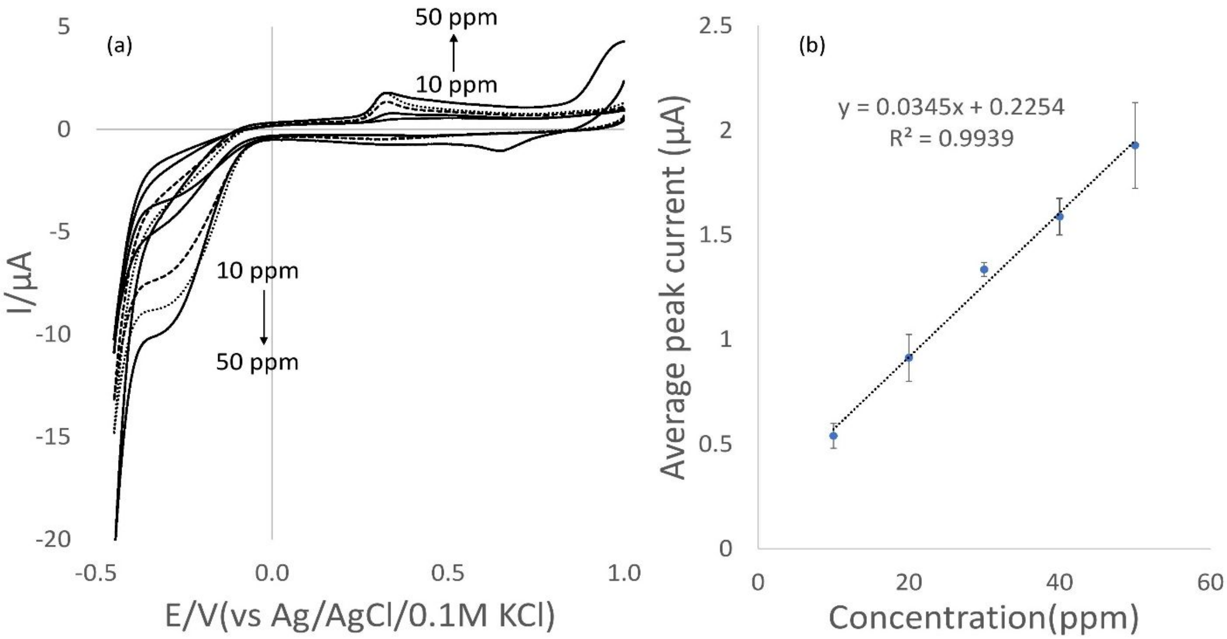

Figure S5. (a) CV Scan of 10 to 50ppm DNT in 2 M H_2SO_4 at scan rate of 50 mV/s using Gold Electrode (aerated). (b) Plot of Anodic Average peak current vs Concentration in ppm of DNT in 2 M H_2SO_4 (n=3).

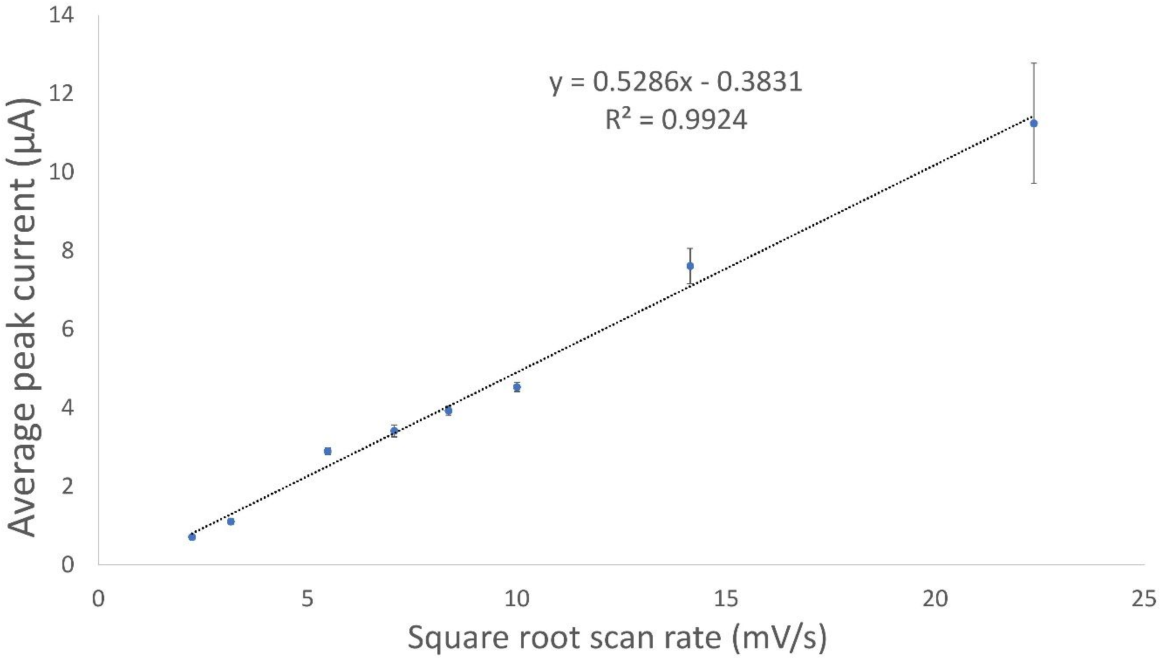

Figure S6. Linear Plot obtained of average anodic peak current vs square root of scan rate (n=3).
